# Inoculation against Black-leg*Adam’s “Wochenschrift fur Thierheilkunde,” 120, 27, 1885.

**Published:** 1885-10

**Authors:** 


### INOCULATION AGAINST BLACK-LEG.*

In seven Swiss Cantons—or districts—there were inoculated 2,199 cattle on account of the prevalence of this disease. These animals were then placed at pasture upon lands where the disease was known to prevail each year to an alarming extent. The results in various localities in the Alps where 908 inoculated and 1,650 non-inoculated head of cattle were pastured were, that of the 908 inoculated animals only two acquired the

* Adam's "Wochenschrift fur Thierheilkunde," 120, 27, 1885.

disease; while of the 1,650 non-inoculated, 101 died, Inoculation has been adopted to a much greater degree on this account. The preparation of material for inoculation was after the method of Arloing, Cornevin and Thomas, and is as follows:

Take a quantity of the blackest appearing tissue from the tumefactions of this disease and cut it up in small pieces; to this is to be added one-half its weight of ordinary water; the mass is then to be rubbed up in a mortar until it becomes a fine mush, it is then to be pressed through a strong linen cloth. This product is now to be filtered and placed in very thin layers in broad, flat-bottomed glass dishes, and quickly dried at a temperature of between 32° and 35° C., then scraped off with the cleanest of knives—the blade should be passed through a flame first—and put into glass tubes and hermatically sealed. For use, take one part of this material—virus—and mix it with two parts of water by rubbing; it is then to be put in a saucer, and placed for seven hours in a thermostat, at at the above temperature. The substance thus obtained has the appearance of brown scales.

Two varieties of virus must be prepared—a primary and secondary.

The first is obtained when the virulent fluid *first* prepared is exposed to 100° C., and the *second* when the same fluid is exposed to 85.6° C.; heat for seven hours in a thermostat. One centigram of the desiccated material corresponds to one gram. fluid before it is introduced into the oven. Such dried material will preserve its active qualities for at least a year.

The end of the tail is the point taken for the inoculation. The animal should be inoculated twice within ten or fourteen days, at first with the secondary and then with the primary virus.

To this end we have fifty parts, by weight, of water, to one of the dried material, and rub them up thoroughly; filter. The amount received is then carefully introduced into the subcutaneous tissue, by first taking a sterilized trochar, and making a canal eight ccms. long, and then introducing the syringe; the skin should be closely compressed upon the trochar so as to prevent the fluid running out. The wound should be carefully covered on removal of the instrument.

The proper time for inoculation is in early spring, while the cold weather continues. Bad results seldom follow. It should not be done after warm weather sets in.

According to Arloing and Cornevin, the inoculated animals retain an immunity against natural infection for from seventeen to eighteen months.

A supreme rule for all such treatment is clean and absolutely sterilized utensils.

